# Low-dose corticosteroid treatment and mortality in refractory abdominal septic shock after emergency laparotomy

**DOI:** 10.1186/s13613-015-0074-8

**Published:** 2015-10-29

**Authors:** Takashi Tagami, Hiroki Matsui, Kiyohide Fushimi, Hideo Yasunaga

**Affiliations:** Department of Clinical Epidemiology and Health Economics, School of Public Health, Graduate School of Medicine, The University of Tokyo, 7-3-1 Hongo, Bunkyo-ku, Tokyo, 113-8555 Japan; Department of Emergency and Critical Care Medicine, Nippon Medical School Tama Nagayama Hospital, Tokyo, 206-8512 Japan; Department of Health Informatics and Policy, Graduate School of Medicine, Tokyo Medical and Dental University, 1-5-45, Yushima, Bunkyo-ku, Tokyo, 113-8510 Japan

**Keywords:** Outcomes assessment, Peritonitis, Pneumonia, Sepsis, Steroid

## Abstract

**Background:**

The role of low-dose corticosteroid as an adjunctive treatment for abdominal septic shock remains controversial.

**Methods:**

We identified refractory septic shock patients who required noradrenaline and at least one of other vasopressor/inotropic (dopamine, dobutamine or vasopressin) following emergency open laparotomy for perforation of the lower intestinal tract between July 2010 and March 2013 using the Japanese Diagnosis Procedure Combination inpatient database. In-hospital mortality was compared between the low-dose corticosteroid and control groups.

**Results:**

There were 2164 eligible patients (155 in the corticosteroid group, 2009 in the control group). We observed no significant difference between the groups in terms of in-hospital mortality in the unadjusted analysis [corticosteroid vs. control groups, 19.4 and 25.1 %, respectively; difference, −5.7 %; 95 % confidence interval (CI), −12.8 to 1.3]; however, a significant difference in in-hospital mortality was evident in the propensity score-weighted analysis (17.6 and 25.0 %, respectively; difference, −7.4 %; 95 % CI −9.9 to −5.0). An instrumental variable analysis with the hospital low-dose corticosteroid prescription proportion showed that receipt of low-dose corticosteroid was significantly associated with reduction in in-hospital mortality (differences, −13.5 %; 95 % CI −24.6 to −2.3).

**Conclusions:**

Low-dose corticosteroid administration may be associated with reduced in-hospital mortality in patients with refractory septic shock following emergency laparotomy for lower intestinal perforation.

## Background

Despite recent developments in the diagnosis and treatment of sepsis, mortality in septic shock patients remains unacceptably high [1–3]. Corticosteroids offer potential as inhibitors of inflammation and in treating adrenal insufficiency and shock reversal; the use of corticosteroids may be useful as an additional therapy for septic shock [1, 4, 5]. The effectiveness of corticosteroids has been repeatedly evaluated using one of the gold standard experimental models for evaluating sepsis, cecal ligation and puncture models [6–9]. However, in clinical practice, there has been long-standing debate about the benefits of low-dose corticosteroid use in sepsis patients, and no consensus has been reached.

Recent landmark trials and meta-analyses of randomized controlled trials have produced conflicting results about the association between low-dose corticosteroid treatment and patient mortality in sepsis [3, 5, 10–20]. However, some meta-analyses have suggested that corticosteroid therapy may more likely benefit patients with severe septic shock that are vasopressor dependent [12, 15, 19]. Thus, the Surviving Sepsis Campaign guidelines recommend considering the use of low-dose corticosteroids for patients with septic shock who have responded poorly to fluid resuscitation and vasopressor agents [1].

A recent multicenter large database study has suggested that the early administration of low-dose corticosteroids was not associated with decreased mortality when they were administered to unselected patients with septic shock [10]. The host response and inflammatory (anti-inflammatory) response mechanism of sepsis vary widely and depend on the following: the initial site of infection; causative organisms; underlying health status of patients; and the status of surgically controlling the infection source [2, 21]. A recent study has also suggested that there is a significantly different mortality according to differences in the infectious organisms and sites [22]. Therefore, when evaluating treatment efficacy of corticosteroids for septic shock, the underlying diseases must be as homogeneous as possible.

We hypothesized that low-dose corticosteroid therapy would be effective for treating refractory septic shock patients after emergency laparotomy for intestinal perforation; this is because the causative insults and pathophysiology may resemble those in cecal ligation and puncture models [6, 23]. The purpose of the present study was to evaluate this hypothesis in a large-scale clinical setting using a nationwide inpatient database.

## Methods

The current study was approved by the Institutional Review Board of The University of Tokyo. The board waived the requirement for informed patient consent because of the anonymous nature of the data.

### Data source and patient selection

The Japanese Diagnosis Procedure Combination (DPC) database was used in the current study [24–27]. The database includes administrative claims and discharge abstract data for all inpatients discharged from more than 1000 hospitals that contribute to the database; the number amounts to 92 % (244/266) of all tertiary-care emergency hospitals in Japan [24–27]. The baseline patient information in the database includes the following: age; sex; primary diagnosis; comorbidities on admission; and post-admission complications coded using the International Classification of Diseases, 10th Revision (ICD-10) codes and written in Japanese. Complications that occurred after admission are clearly differentiated from comorbidities that were already present on admission (on day 0). The DPC database also includes the dosages and dates of all drugs and blood products administered during hospitalization. All interventional and surgical procedures are coded using original Japanese codes. The dates of hospital admission and discharge, emergency laparotomy, bedside procedures, drugs administered, and discharge status (alive or deceased) are recorded using a uniform data submission format. To optimize the accuracy of the recorded diagnoses, the responsible physician is obliged to record the diagnoses with reference to medical charts. In addition, the diagnostic records are linked to a payment system, and attending physicians are required to report objective evidence in disease diagnosis for reimbursement of treatment [24–27].

### Patient selection

We identified septic shock patients who required noradrenaline and one or more other vasopressor/inotropic agents after emergency laparotomy for perforation of the lower intestinal tract from July 1, 2010 to March 31, 2013. The inclusion criteria for this analysis were as follows: (1) age ≥15 years; (2) confirmed diagnosis of perforation of the lower gastrointestinal tract on admission (coded in the primary diagnosis or among comorbidities in the admission section of the database); (3) having undergone open abdominal emergency laparotomy—except exploratory laparotomy—on days 0 or 1; (4) refractory septic shock, defined as the use of at least two vasopressor or inotropic agents, including noradrenaline (i.e., noradrenaline and at least one of the following—dopamine, dobutamine, or vasopressin); and (5) antibiotic administration on days 0 or 1. The following patients were excluded: (1) those with coexisting diseases requiring routine corticosteroid treatment (i.e., chronic obstructive pulmonary disease, sarcoidosis, asthma, inflammatory bowel disease, pemphigus, pemphigoid, connective tissue disease, or angiitis); (2) patients discharged within 2 days after admission (i.e., who died within 2 days [28]; this was to avoid immortal time bias); (3) those who received low-dose corticosteroids after day 2; and (4) patients who received high-dose corticosteroids [29]. Low-dose corticosteroid treatment was defined as intravenous infusion of 500 mg of hydrocortisone (or an equivalent dose of dexamethasone, methylprednisolone, prednisolone, or betamethasone); any greater dose was defined as high-dose corticosteroid therapy [20].

### Variables and end point

In addition to the baseline characteristics on admission, several other variables were evaluated in the current study. The Japan Coma Scale score, which is used to assess consciousness level and correlates well with the Glasgow Coma Scale, was recorded in all patients on admission and was used to categorize the patients into four groups: 0 (alert); 1–3 (delirium); 10–30 (somnolence); and 100–300 (coma) [24–27]. Hospitals were categorized as academic or nonacademic institutions. Hospital volume was defined as the number of eligible patients treated at each hospital and was subcategorized into tertiles. The main end point was all-cause in-hospital mortality. The other endpoints were catecholamine-free days and ventilator-free days [30]. Catecholamine- and ventilator-free days were defined as the number of days alive without catecholamine/mechanical ventilation assistance, respectively, during the first 28 days after admission, and patients who died before day 28 were assigned zero [30].

### Statistical analysis

#### Propensity score analysis

Descriptive statistics are presented for all patients and propensity score-weighted [inverse probability of treatment (IPT) weighting] groups. Based on the estimated propensity scores for each patient, we performed IPT-weighted analyses for the low-dose corticosteroid and control groups [31–33]. To estimate the propensity score, we fitted a logistic regression model for undergoing low-dose corticosteroid treatment as a function of patient demographics and medication or interventions performed on days 0 or 1. We included the following: age; sex; hospital type (academic or nonacademic) and volume; consciousness level; coexisting diseases; blood culture test performed; intermittent hemodialysis or continuous renal replacement therapy after admission; postoperative polymyxin B hemoperfusion; type of catecholamine used; vasopressin used; initial use of two or more antibiotics and each type of initial antibiotic administered; drugs for disseminated intravascular coagulation (antithrombin, thrombomodulin, gabexate mesilate, nafamostat mesilate, or ulinastatin); use of intravenous immunoglobulin, albumin, or sivelestat sodium; and blood transfusion [24–27, 33]. We used IPT as the weights based on the propensity score; we did so to create a synthetic sample, in which the distribution of measured baseline covariates was independent of treatment assignment [31–33]. We defined the weights as follows: W_*i*_ = Z_*i*_/e_*i*_ + (1 − Z_*i*_)/1 − e_*i*_, where Z_*i*_ was an indicator variable denoting whether or not the *i*th subject was treated (Z_*i*_ = 1) or untreated (Z_*i*_ = 0); e_*i*_ signified the propensity score for the *i*th subject. An essential component in any propensity score analysis is assessing the similarity of baseline covariates between treated and untreated subjects in the sample weighted by IPT [31–33]. We examined the balance in baseline variables using standardized differences, where >0.10 was regarded as imbalanced [31–33]. We compared the categorical variables using the Chi-square test or Fisher’s exact test.

#### Instrumental variable analysis

In addition, we conducted instrumental variable analysis as a confirmatory analysis for the propensity score analyses [34]. We used the proportion of low-dose corticosteroid use at each hospital as an instrumental variable, and we computed the difference in in-hospital mortality between the groups with and without corticosteroid treatment. We implemented this approach using a two-stage least-squares regression, which also adjusted for patient demographic characteristics. We classified hospitals that administered corticosteroid to the 90th percentile or more of eligible patients as hospitals with a preference for low-dose corticosteroid use; hospitals that administered low-dose corticosteroid to less than the 90th percentile of eligible patients were classed as hospitals without a preference for low-dose corticosteroid treatment. We estimated the risk difference and 95 % confidence interval (CI) by means of the ivreg2 procedure using Stata/SE 13.0 (Stata Corp., College Station, TX, USA). To confirm that the proportion of hospital low-dose corticosteroid treatment was not a weak instrument, we used a partial *F* test [34]. The null hypothesis was that there was no association between the proportion of hospital corticosteroid use and actual corticosteroid use. An *F*-statistic greater than 10 suggests that the instrument is not weak [34]. All statistical analyses were performed with IBM SPSS version 22 (IBM Corp., Armonk, NY, USA) and Stata/SE 13.0.

## Results

### Patients

**Fig. 1:**
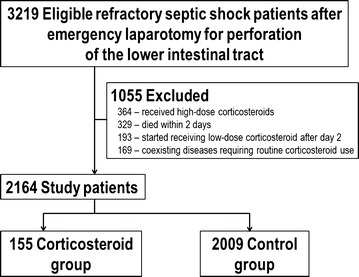
Patient selection

We identified 2164 patients during the 33-month study period as eligible subjects. The patients were divided into the low-dose corticosteroid group (*n* = 155) and control group (*n* = 2009) (Fig. 1). Tables 1 and 2 show the baseline characteristics and treatment of the unadjusted and propensity score-weighted groups. A comparison of the unadjusted groups indicated that patients were more likely to receive low-dose corticosteroid treatment if they required more vasopressin, more carbapenem, or blood transfusion. After propensity score weighting, the baseline patient characteristics were well balanced between the groups, i.e., standardized differences <0.10 for all variables. The mean amount of corticosteroid administered to survivors was 216 ± 11 mg/day of hydrocortisone for 2.5 ± 0.2 days; among non-survivors, it was 218 ± 23 mg/day of hydrocortisone for 3.5 ± 1.2 days.

**Table 1:** Baseline patient characteristics in the unmatched and propensity score-matched groups

Characteristics	Unmatched					Propensity-score matched				
	Control, *n* = 2009		Corticosteroid, *n* = 155		Standardized, difference, %	Control, *n* = 2164		Corticosteroid, *n* = 2101		Standardized difference, %
Age (year)	74.8	(11.5)	75.1	(10.6)	−2.2	74.8	(11.5)	75.5	(10.1)	−5.9
Sex (male)	1049	(52.2)	62	(40.0)	24.7	1111	(51.3)	1009	(48.0)	6.6
Hospital type (academic)	489	(24.3)	42	(27.1)	−6.3	532	(24.6)	583	(27.7)	−7.2
Hospital volume, cases					0.0					
Low, 1–4	673	(33.5)	52	(33.5)	−0.1	725	(33.5)	659	(31.4)	4.6
Medium, 5–9	736	(36.6)	71	(45.8)	−18.7	807	(37.3)	821	(39.1)	−3.6
High, 10–	600	(29.9)	32	(20.6)	21.3	632	(29.2)	622	(29.6)	−0.8
Coexisting disease										
Diabetes	221	(11.0)	17	(11.0)	0.1	238	(11.0)	200	(9.5)	4.9
Old myocardial infarction	42	(2.1)	1	(0.6)	12.5	43	(2.0)	27	(1.3)	5.5
Pneumonia	129	(6.4)	9	(5.8)	2.6	138	(6.4)	139	(6.6)	−1.0
Chronic renal failure	161	(8.0)	7	(4.5)	14.5	168	(7.8)	117	(5.6)	8.8
Disseminated intravascular coagulation	959	(47.7)	81	(52.3)	−9.1	1040	(48.1)	1065	(50.7)	−5.3
Consciousness level (JCS score)					0.0					
Alert	1385	(68.9)	108	(69.7)	−1.6	1493	(69.0)	1367	(65.1)	8.4
Delirium	343	(17.1)	24	(15.5)	4.3	367	(17.0)	391	(18.6)	−4.3
Somnolence	116	(5.8)	10	(6.5)	−2.8	126	(5.8)	168	(8.0)	−8.6
Coma	165	(8.2)	13	(8.4)	−0.6	178	(8.2)	175	(8.3)	−0.4

*IVIG* intravenous immunoglobulin, *JCS* Japan Coma Scale

**Table 2:** Medications and interventions performed on the day 0 or 1 in the unmatched and propensity-matched groups

Variable	Unadjusted					Propensity-score weighted				
	Control, *n* = 2009		Corticosteroid, *n* = 155		Standardized difference, %	Control, *n* = 2164		Corticosteroid, *n* = 2101		Standardized difference, %
Blood culture taken	911	(45.3)	76	(49.0)	−7.4	987	(45.6)	1005	(47.8)	−4.5
Mechanical ventilation	1675	(83.4)	130	(83.9)	−1.3	1805	(83.4)	1752	(83.4)	0.1
Intermittent hemodialysis	255	(12.7)	15	(9.7)	9.6	270	(12.5)	229	(10.9)	4.9
Continuous renal replacement therapy	478	(23.8)	31	(20.0)	9.2	509	(23.5)	487	(23.2)	0.8
PMX	515	(25.6)	35	(22.6)	7.1	550	(25.4)	530	(25.2)	0.5
Catecholamines										
Dopamine use	1820	(90.6)	128	(82.6)	23.7	1949	(90.1)	1895	(90.2)	−0.4
Dobutamine use	386	(19.2)	33	(21.3)	−5.2	419	(19.4)	449	(21.4)	−5.0
Vasopressin	158	(7.9)	27	(17.4)	−29.1	184	(8.5)	154	(7.3)	4.4
Initial antibiotic use										
Initial use of two or more antibiotics	483	(24.0)	38	(24.5)	−1.1	521	(24.1)	556	(26.5)	−5.5
Tazobactam/piperacillin or sulbactam/cefoperazone sodium	180	(9.0)	10	(6.5)	9.4	190	(8.8)	214	(10.2)	−4.8
First-generation cephalosporin	93	(4.6)	4	(2.6)	11.0	97	(4.5)	116	(5.5)	−4.8
Second-generation cephalosporin	758	(37.7)	56	(36.1)	3.3	814	(37.6)	810	(38.5)	−1.9
Third-generation cephalosporin	63	(3.1)	5	(3.2)	−0.5	68	(3.1)	56	(2.7)	2.8
Fourth-generation cephalosporin	46	(2.3)	3	(1.9)	2.5	49	(2.3)	39	(1.9)	2.9
Carbapenem	1248	(62.1)	111	(71.6)	−20.3	1359	(62.8)	1357	(64.6)	−3.7
Antifungal drug	34	(1.7)	3	(1.9)	−1.8	37	(1.7)	41	(2.0)	−1.8
Antithrombin	646	(32.2)	57	(36.8)	−9.7	703	(32.5)	740	(35.2)	−5.8
rhTM	454	(22.6)	38	(24.5)	−4.5	492	(22.7)	543	(25.8)	−7.2
Gabexate mesilate	517	(25.7)	40	(25.8)	−0.2	557	(25.7)	551	(26.2)	−1.1
Nafamostat mesilate	947	(47.1)	72	(46.5)	1.4	1019	(47.1)	1006	(47.9)	−1.6
Ulinastatin	545	(27.1)	39	(25.2)	4.5	584	(27.0)	493	(23.5)	8.1
Sivelestat sodium	735	(36.6)	51	(32.9)	7.7	786	(36.3)	715	(34.0)	4.8
Immunoglobulin	856	(42.6)	61	(39.4)	6.6	917	(42.4)	845	(40.2)	4.4
Albumin	1614	(80.3)	129	(83.2)	−7.5	1743	(80.5)	1761	(83.8)	−8.6
Blood transfusion										
Red blood cells	1094	(54.5)	88	(56.8)	−4.7	1182	(54.6)	1196	(56.9)	−4.6
Fresh frozen plasma	1065	(53.0)	89	(57.4)	−8.9	1154	(53.3)	1090	(51.9)	2.9
Platelets	254	(12.6)	28	(18.1)	−15.1	282	(13.0)	256	(12.2)	2.6
Amount of first blood transfusion										
No. transfusions	833	(41.5)	55	(35.5)	12.3	887	(41.0)	796	(37.9)	6.4
<500 mL	444	(22.1)	41	(26.5)	−10.2	486	(22.5)	535	(25.5)	−7.0
501–1000 mL	556	(27.7)	45	(29.0)	−3.0	601	(27.8)	556	(26.5)	2.9
>1001 mL	176	(8.8)	14	(9.0)	−1.0	190	(8.8)	214	(10.2)	−4.8

*PMX* polymyxin B hemoperfusion, *rhTM* recombinant human soluble thrombomodulin

### End points

Although the in-hospital mortality did not significantly differ between the corticosteroid and control groups in the unadjusted analysis [19.4 %, 30/155 vs. 25.1 %, 504/2009; difference, −5.7 %; 95 % confidence interval (CI), −12.8 to 1.3], a significant difference existed in the propensity score-weighted analysis (17.6 %, 369/2101 vs. 25.0 %, 541/2164; difference, −12.4 %; 95 % CI −22.0 to −2.7) (Table 3). In the instrumental variable model, the null hypothesis that there was no association between the proportion of hospital corticosteroid use and actual corticosteroid use was rejected (*p* < 0.001; *F*-statistic, 702). The estimated reduction in in-hospital mortality associated with receipt of corticosteroid was 13.5 % (95 % CI −24.6 to −2.3).

**Table 3:** Comparisons of outcomes between groups

Groups	Corticosteroid	Control	Difference (95 % CI)
Unadjusted groups			
In-hospital mortality	19.4 % (30/155)	25.1 % (504/2009)	−5.7 % (−12.8 to 1.3)
Catecholamine-free days (SD)	18.9 (9.9)	17.4 (10.5)	1.5 (−0.2 to 3.2)
Ventilator-free days (SD)	18.7 (10.5)	17.4 (10.7)	1.2 (−0.5 to 3.0)
Propensity score-weighted groups			
In-hospital mortality	17.6 % (369/2101)	25.0 % (541/2164)	−7.4 % (−9.9 to −5.0)
Catecholamine-free days (SD)	19.3 (9.6)	17.4 (10.5)	1.9 (1.3 to 2.5)
Ventilator-free days (SD)	19.1 (10.4)	17.4 (10.7)	1.7 (1.1 to 2.4)

No significant difference in the number of catecholamine-free days was documented in the corticosteroid and control groups for unadjusted patients (18.9 vs. 17.4; difference, 1.5; 95 % CI −0.2 to 3.2); however, more catecholamine-free days were observed in the corticosteroid group among the propensity score-weighted groups (19.3 vs. 17.4 days; difference, 1.9; 95 % CI 1.3–2.5). Similarly, no significant difference in the number of ventilator-free days was found in the corticosteroid and control groups for unadjusted patients (18.7 vs. 17.4; difference, 1.2; 95 % CI −0.5 to 3.0); however, more ventilator-free days were documented in the corticosteroid group among the propensity score-weighted groups (19.1 vs. 17.4 days; difference, 1.7; 95 % CI 1.1–2.4).

## Discussion

In this study, using data from a nationwide database, we performed propensity score and instrumental variable analyses of 2164 refractory septic shock patients following emergency laparotomy for intestinal perforation. The results suggest that there may be a significant association between corticosteroid use and reduction in in-hospital mortality for these severe septic patients. Additionally, corticosteroid use was associated with more ventilator- and catecholamine-free days with these patients.

The use of low-dose corticosteroids as an adjunctive therapy in septic shock has been controversial for decades. Several experimental studies have indicated that corticosteroids were effective for treating abdominal sepsis in a cecal ligation and puncture model [7, 8]. Although significant differences may exist between experimental models and the real-world clinical setting [6], the pathophysiology of the eligible septic patients in the present study may resemble that in a cecal ligation and puncture model. Several experimental and clinical findings suggest that glucocorticoid may play an important role in counteracting critical illness-related corticosteroid insufficiency—defined as inadequate corticosteroid activity relative to the severity of illness [35]. However, owing to a lack of clinical data, the latest Surviving Sepsis Campaign advises against using intravenous hydrocortisone for treating adult septic shock patients if adequate fluid resuscitation and vasopressor therapy are able to restore hemodynamic stability [1]. Moreover, a recent study by Contrael et al. [36] has indicated that significant variability was observed when corticosteroids were prescribed for septic shock; thus, the decision to prescribe low-dose corticosteroids in septic shock must have depended on the physicians’ clinical experience. In addition, low-dose corticosteroids are commonly administered globally in treating any type of septic shock without considering the underlying infection site or pathophysiology of the cause of sepsis [18].

A recent multicenter large database study has suggested that the early administration of low-dose corticosteroids was not associated with decreased mortality when they were administered to unselected patients with septic shock in general [10]. The host response and inflammatory (anti-inflammatory) response mechanism of sepsis show wide variety and depend on the following: the initial site of infection (e.g., lungs vs. abdomen); causative organisms (e.g., gram-positive vs. gram-negative); patterns of acute organ dysfunction (e.g., primary vs. secondary acute respiratory distress failure); underlying health status of the patients (i.e., age or coexisting disease); and surgical control of the infection source [2, 21]. We thus believed that the underlying pathophysiology must be as homogeneous as possible when evaluating treatment efficacy of corticosteroids for septic shock. In addition, previous studies have suggested that there may be a beneficial effect of low-dose corticosteroids for sepsis on mortality in patients with the highest severity of illness, such as refractory septic shock [10, 27].

One strength of the present study was including over 2000 patients throughout Japan to evaluate the effect of corticosteroids in refractory septic shock after emergency laparotomy for intestinal perforation. Most eligible patients presumably sought medical care and were admitted to hospitals immediately after the onset of the perforation compared with patients with other sepsis-related diseases (e.g., pneumonia, urinary tract infection or cholecystitis), because such patients typically present with an acute onset of abdominal pain that is persistent, progressive, and unremitting due to perforation of intestine and subsequent peritonitis. We selected only patients who had undergone an emergency operation that finished at an appropriate time (i.e., the operation was completed on day 0 or 1) and received antibiotics. In the current cohort, the causative insults of sepsis must have been homogeneously enteric bacteria. In fact, the major initial antibiotics given to the patients were carbapenem or second-generation cephalosporin. In addition, two or more antibiotics were used in 24 % of the cases. Antibiotics with a broad-spectrum regimen and adequate coverage against typical causative organisms (i.e., enteric bacteria) are recommended for use as empiric therapy in septic shock patients according to current sepsis guidelines [1].

In the present study, the analysis of the baseline patient characteristics in the unmatched group showed more corticosteroid use in patients with more severe illness; however, the propensity score weighting successfully balanced the characteristics between the patient groups with and without corticosteroid use, including factors that have the potential to affect mortality or are known to affect mortality in patients with sepsis. The results of the propensity score-weighted analysis suggested that refractory septic shock patients who were prescribed corticosteroids were less likely to die than those who were not. Moreover, to overcome the bias of unmeasured confounding factors, we also performed an instrumental variable analysis and confirmed the robustness of our results. Although neither propensity score analysis nor instrumental variable analysis is perfect for a robust assessment, consistent results from instrumental variable analysis may serve as a useful confirmatory analysis for propensity score analyses. International prospective trials are needed to confirm the effect of low-dose corticosteroids on refractory septic shock; however, currently, such trials are not easy to implement, e.g., a very slow inclusion rate with the CORTICUS study [17]. Before such trials can be undertaken, it is therefore necessary to have well-analyzed retrospective studies that permit sound hypotheses to be generated. We regard the present study as part of this kind of initial effort to acquire basic data. The findings of this investigation will help advance our understanding of the pathophysiology of septic shock.

Several limitations should be considered when interpreting the results of this study. First, this investigation was conducted retrospectively in an observational manner without randomization. Although a propensity score analysis was used to adjust for differences in baseline characteristics and disease severity, bias could still have been present in the form of unmeasured confounders. Important examples of such confounders are the Acute Physiology and Chronic Health Evaluation score, ventilator settings, and extravascular lung water [37]. However, the data related to those confounders were not available in the DPC database. We therefore validated our results using instrumental variable analysis to compensate for these potential unmeasured confounders. Second, this study focused on refractory septic shock patients following emergency laparotomy for lower intestinal perforation. Thus, the results cannot be generalized to other cause of sepsis (e.g., pneumonia, meningitis, urinary tract, and soft tissue infections). Further studies are required to investigate which specific population has a better outcome in the case of severe sepsis. Third, it is very important to evaluate whether steroids were given continuously and administered with or without tapering; it is also necessary to determine the value of cortisol levels. However, because of our retrospective study design, we were unable to assess those variables. Fourth, only 155 of 2164 (7 %) of the eligible patients received steroids, which suggests that only the sickest patients obtained that treatment. The small numbers in the treatment group prevented us from performing propensity score-matched analyses in this study, i.e., most cases were not evaluated if matching was undertaken. One of the strengths of the IPT weighting method is that data from all patients are used [38]; however, treated subjects with a very low propensity score or untreated subjects with a high propensity score are accorded high weights. This may be a significant concern if the study group is heterogeneous. In the current study, though, we attempted to select a homogeneous patient group by utilizing strict inclusion and exclusion criteria: all the patients had adult refractory septic shock following open abdominal emergency laparotomy (i.e., the causative insults and pathophysiology could have resembled those in cecal ligation and puncture models). Fifth, several treatments listed in Table 2 (e.g., frequent use of polymyxin B hemoperfusion, antithrombin, recombinant human soluble thrombomodulin, intravenous immunoglobulin, and carbapenems) are uncommonly used outside Japan. However, to the best of our knowledge, there is no evidence to suggest that those treatments exert a synergetic effect with steroids. Rather, those factors are important confounders when evaluating the effect of low-dose steroids for treating septic shock. We therefore used those confounding factors to determine the propensity score and also adjusted for them in the instrumental variable analysis.

## Conclusions

This nationwide retrospective study found that low-dose corticosteroid use may be associated with better prognosis in patients with refractory septic shock following emergency laparotomy for lower intestinal perforation. Future large, multinational, randomized trials are required to confirm our results.
